# Expression analysis of human pterygium shows a predominance of conjunctival and limbal markers and genes associated with cell migration

**Published:** 2009-11-20

**Authors:** C.J. Jaworski, M. Aryankalayil-John, M.M. Campos, R.N. Fariss, J. Rowsey, N. Agarwalla, T.W. Reid, N. Dushku, C.A. Cox, D. Carper, G. Wistow

**Affiliations:** 1 Section on Molecular Therapeutics, NEI/NIH, Bethesda, MD; 2 National Cancer Institute, Bethesda, MD; 3 NEI-Biological Imaging Core, NEI/NIH, Bethesda, MD; 4 Ophthalmology and Visual Sciences and Cell Biology and Biochemistry, Texas Tech University Health Sciences Center, Lubbock, TX; 5 Kaiser Permanente Medical Center, Sacramento, CA; 6 John A. Burns School of Medicine, University of Hawaii, Honolulu, HI; 7 Section on Molecular Structure and Functional Genomics, NEI/NIH, Bethesda, MD

## Abstract

**Purpose:**

Pterygium is a vision-impairing fibrovascular lesion that grows across the corneal surface and is associated with sunlight exposure. To increase our understanding of the cells types involved in pterygium, we have used expressed sequence tag analysis to examine the transcriptional repertoire of isolated pterygium and to identify marker genes for tissue origin and cell migration.

**Methods:**

An unnormalized unamplified cDNA library was prepared from 15 pooled specimens of surgically removed pterygia as part of the NEIBank project. Gene expression patterns were compared with existing data for human cornea, limbus, and conjunctiva, and expression of selected genes was verified by immunofluorescence localization in normal eye ocular surface and in pterygium.

**Results:**

Sequence analysis of 2,976 randomly selected clones produced over 1,800 unique clusters, potentially representing single genes. The most abundant complementary DNAs from pterygium include clusterin, keratins 13 (*Krt13*) and 4 (*Krt4*), S100A9/calgranulin B, and spermidine/spermine N1-acetyltransferase (*SAT1*). Markers for both conjunctiva (such as keratin 13/4 and *AQP3*) and corneal epithelium (such as keratin 12/3 and *AQP5*) were present. Immunofluorescence of Krt12 and 13 in the normal ocular surface showed specificity of Krt12 in cornea and Krt13 in conjunctival and limbal epithelia, with a fairly sharp boundary at the limbal–corneal border. In the pterygium there was a patchy distribution of both Krt12 and 13 up to a normal corneal epithelial region specific for Krt12. Immunoglobulins were also among the prominently expressed transcripts. Several of the genes expressed most abundantly in excised pterygium, particularly *S100A9* and *SAT1*, have roles in cell migration. *SAT1* exerts its effects through control of polyamine levels. IPENSpm, a polyamine analogue, showed a significant ability to reduce migration in primary cultures of pterygium. A number of genes highly expressed in cornea were not found in pterygium (several small leucine-rich proteoglycan family members) or were expressed at considerably lower levels (*ALDH3A1* and decorin).

**Conclusions:**

The expression pattern of keratins and other markers in pterygium most closely resemble those of conjunctival and limbal cells; some corneal markers are present, notably Krt12, but at lower levels than equivalent conjunctival markers. Our data are consistent with the model of pterygium developing from the migration of conjunctival- and limbal-like cells into corneal epithelium. Identification of genes with roles in cell migration suggests potential therapeutic targets. In particular, the ability of polyamine analogues to reduce migration in primary cultures of pterygium presents a possible approach to slowing pterygium growth.

## Introduction

Pterygium is an overgrowth of fibrovascular tissue, often with a wing-like appearance, from the conjunctiva over the cornea [1-4]. It typically induces astigmatism [5,6], which can be visually significant. Untreated it can invade the cornea, occluding the pupil and leading to loss of vision. Pterygium is common in many parts of the world and is particularly prevalent in equatorial regions and high altitudes, implicating UV exposure as a triggering insult. The principal treatment for pterygium is surgical removal. This approach can have high success rates, but recurrence requiring repeat surgery and complications occur. Conjunctival autografts, amniotic membrane transplantation, and treatment with radiation or chemotherapeutic agents, usually mitomycin C, are often employed in attempts to reduce recurrence (for review, see [7]). Pterygium pathophysiology is characterized by invasion of the basement membrane of normal cornea with the concomitant dissolution of Bowman’s layer [8]. In accordance with this, pterygiuim cells show elevated expression of matrix metalloproteinases (MMPs) [9].

Studies aimed at characterization of the molecular composition of pterygium have been reported. Microarray comparisons of pterygium and conjunctiva-derived fibroblasts found evidence for increased expression of insulin-like growth factor-binding protein 2 in pterygium [2]. Another microarray study compared human whole pterygium and autologous conjunctiva and found increased levels of extracellular-matrix-related, proinflammatory, angiogenic, fibrogenic, and oncogenic transcripts, including MMPs, fibronectin, macrophage inflammatory protein 4 (MIP-4), and lipocalin 2 in pterygium [10]. A more recent microarray analysis by Tong et al. [11] compared expression of primary pterygia, recurrent pterygia, and uninvolved conjunctiva. Finding increased expression of adhesion molecules and extracellular matrix and structural proteins (fibronectin; collagen and keratin family members), they concluded that aberrant wound healing processes play a role in pterygium pathogenesis. Epithelial-mesenchymal transition (EMT), with downregulation of E-cadherin and upregulation of β-catenin and lymphoid-enhancer-factor-1, has also been proposed as a mechanism for the origin of pterygial fibroblasts [1,12]. Pterygium has additionally been described as a benign neoplastic lesion [9,13,14].

For another view of the transcriptional repertoire of pterygium, we have used expressed sequence tag (EST) analysis of an unnormalized unamplified complementary DNA (cDNA) library made from pooled, postmortem pterygium through the NEIBank project for ocular genomics [15,16]. The results show abundant expression of differentiated markers for both conjunctiva and corneal epithelium, with conjunctival markers predominating. Other abundant transcripts are associated with cell migration and suggest new avenues for investigating possible therapeutic approaches.

## Methods

### Tissue procurement and complementary DNA library construction

For library construction, 15 postmortem pterygia were excised and immersed in RNA*later* (Ambion, Austin, TX). The procedure for obtaining the tissues was within the tenets of the Declaration of Helsinki. Pterygia were pooled and total RNA was extracted using RNAzol following the manufacturer’s protocol (Tel-Test Inc., Friendswood, TX). Total RNA (80 µg) was used for library construction.

Poly(A)+ RNA was prepared from the total RNA using an oligo-dT cellulose affinity column (Invitrogen, Carlsbad, CA). Oligo-dT-primed cDNA was synthesized at Bioserve Biotechnology (Laurel, MD) using the Superscript II system (Invitrogen, Carlsbad, CA), as described previously [17]. The cDNA was run over a resin column (Sephacryl S-500 HR; Invitrogen) to fractionate cDNA larger than 500 bp. The first two 35-µl fractions, containing cDNA, were pooled and directionally cloned in Not I/Sal I sites in the pCMVSPORT6 vector (Invitrogen) to make a cDNA library with internal NEIBank code designation *nav*. The library consisted of approximately 10^7^ primary recombinants. The average insert size was estimated to be 1.63 kb; insert sizes ranged from 0.4 kb to 2.7 kb.

### Complementary DNA sequencing and bioinformatics

Approximately 3,000 clones were randomly selected and sequenced from the 5' end at the National Institutes of Health Intramural Sequencing Center. Grouping and identification of sequence tags (GRIST) was used to analyze and assemble the data and to display the results in Web-page format [17]. Sequences were also examined using SeqMan II (DNAstar, Madison, WI) to check assembly of clusters and to examine alternative transcripts. Pterygium data were compared with EST data for other relevant datasets, using the NEIBank library comparison tool.

### Pathway analysis

Pathway analysis was performed using the software provided by Ingenuity Pathway Analysis (IPA; Ingenuity, Mountain View, CA). Identifiers for genes represented by three or more clones in the dataset were uploaded. To simplify the view, genes of the protein translational machinery (ribosomal proteins, elongation, and initiation factors), which are abundant in most cDNA libraries, were omitted. A network based upon the Ingenuity pathways knowledge base was generated by connecting protein nodes to represent direct and indirect biological relationships. Unconnected nodes were removed. Functional analysis of the network was performed on 51 connected genes/proteins.

### Tissue collection and preparation for immunofluorescence

Human eyes obtained from the Southern Eye Bank (Metairie, LA) were treated according to the Helsinki Statement for the Use of Human Tissue. Upon arrival, eyes were fixed by immersion in 4% paraformaldehyde in phosphate buffered saline, pH 7.3, overnight and transferred to PBS. Anterior segments were then removed to separate the nasal side together with sclera, conjunctiva, cornea, and pterygia. The tissue sections were oriented and embedded in Tissue-Tek optimum cutting temperature (OCT) compound (Sakura Finetek U.S.A., Inc., Torrance, CA), stored at -70 ºC, and cut into horizontal 10-µm-thick sections.

For confocal immunolocalization studies, 5% normal serum was used as the blocking reagent for 30 min at 23 ºC, followed by incubation for 1 h with the following primary antibodies: rabbit anti-cytokeratin 13 (polyclonal, 1:50; Protein Group, Inc., Chicago, IL), mouse anti-cytokeratin 12 (monoclonal, 1:25; Biogenesis, Poole, UK), mouse anti-human clusterin (monoclonal, 1:50; BD Bioscience, San Jose, CA), mouse anti-S100A6 (calcyclin; monoclonal 1:1,000; Sigma®, Saint Louis, MO), mouse anti-SAT (spermidine/spermine N1 acetyltransferase; polyclonal, 1:200), mouse-anti-annexin II (3D5/4; monoclonal, 1:50; Gene-Tex Inc., San Antonio, TX).

Sections were washed in immunolabeling buffer (PBS 1X containing 0.5% bovine serum, 0.2% Tween-20, and 0.05% sodium azide) then incubated for 30 min in the following fluorochrome-conjugated secondary antibodies (goat anti-mouse Alexa Fluor® 488, goat anti-rabbit Alexa Fluor® 568, and 4',6-diamidino-2-phenylindole [DAPI; Molecular Probes, Carlsbad, CA]). Primary antibodies were omitted from sections used as negative controls. Sections of labeled human anterior segment were washed, mounted in Gel-Mount (Biomeda, Foster City, CA), and cover-slipped. A Leica SP2 confocal microscope (Leica Microsystems, Exton, PA) was used to image samples.

### Cell culture

Use of pterygium specimens was approved by the Institutional Review Board of Texas Tech School of Medicine, Lubbock, TX. Specimens were handled in accordance with the Declaration of Helsinki. Pterygium were collected at surgery, and the epithelium was separated from the underlying stroma. The epithelial tissue was held in place with a cover slip for 3 days in Dulbecco's Modified Eagle Medium Nutrient Mixture F-12 (DMEM/F12; Invitrogen, Carlsbad, CA) medium supplemented with 10% fetal calf serum (FCS), 200 mM L-glutamine, 0.5% dimethyl sulfoxide (DMSO), and 1% antibiotic/antimycotic, during which time the cells migrated from the explant. The explant was then removed and the medium changed to kerotinocyte- serum free medium with 5% FCS and 1% antibiotic/antimycotic to further promote epithelial cell growth. When the cells were 60–80% confluent, they were passaged several times with 0.25% trypsin. The cells were then placed in DMEM/F12 medium supplemented with 5% fetal bovine serum, 1% L-glutamine, 0.5% DMSO, and 1% antibiotic/antimycotic. The medium was renewed every 2 to 3 days. Passage 5 cells were used in our experimentation.

### Cell migration assay

To analyze pterygium cell migration, we used the CytoSelect 96-well cell migration assay (8 μm, Fluorometric Format; Cell Biolabs, San Diego, CA), following the manufacturer’s instructions. Briefly, feeder tray wells were filled with 150 μl medium (±serum). Confluent cells, grown to passage 5, were harvested; 100-μl aliquots of serum-free medium containing 0.1–1.0×10^6^ cells/ml were added to the wells of the membrane tray (upper tray) with or without polyamine analogues. Migration assays were run for 18 h at 37 °C in a 5% CO_2_ incubator, after which medium was removed from the membrane tray and the tray was placed over a harvesting tray containing 150 μl of the proprietary cell detachment solution for 30 min at 37 °C. The cells were treated with fluorescent dye and measured at 480/520 nm using a Victor3 multi-label reader (PerkinElmer, Shelton, CT). Initial experiments showed that the extent of migration was directly related to the amount of serum added to the bottom chamber of the assay.

### Polyamine analogues

Various concentrations (0–10 μM) of N1,N11-diethylnorspermine (BENSpm) and (S)-N1-(2-methyl-1-butyl)-N11-ethyl-4,8-diazaundecane (IPENSpm) were tested in the migration assay by including various concentrations (0–10 μM) in the wells of the upper tray. Toxicity of the compounds at 10-μM concentrations was checked by the Cell Counting Kit-8 from Dojindo (Gaithersburg, MD).

## Results and discussion

After vector trimming and removal of mitochondrial and contaminant sequences, almost 3,000 clones yielded 2,424 high-quality sequence reads for EST analysis. GRIST assembled these clones into 1,832 unique clusters of which 87% consisted of single clones. Ninety clusters contained three or more clones. Since this is an unnormalized library it provides a view of the native abundance of transcripts in pterygium. Table 1 lists the 25 most abundant transcripts (five or more clones) and indicates those for which the literature describes preferential expression in corneal epithelium, conjunctiva, or limbus. The entire dataset is available at the NEIBank website, with the designation NbLib0106 NEI human pterygium unnormalized (nav).

**Table 1:** The 25 most abundantly represented genes in the pterygium cDNA library.

**Rank**	**Symbol**	**Description**	**Number of clones**	**Conjunctiva**	**Limbus**	**Cornea**
1	*EEF1A1*	eukaryotic translation elongation factor 1a1	22			
2	*CLU*	clusterin	14			
3	*KRT13*	keratin 13	13	X	X	
4	*IGKV1-5*	kappaM immunoglobulin light chain	13			
5	*S100A9*	S100 calcium binding protein A9	10			
6	*SAT1*	spermidine/spermine N1-acetyltransferase 1	9			
7	*KRT4*	keratin 4	9	X	X	
8	*KRT19*	keratin 19	8			
9	*UPLP*	Uroplakin-like protein	7			
10	*ANXA2*	annexin A2	7			
11	*ALDH3A1*	aldehyde dehydrogenase 3 A1	7			X
12	*EIF4G2*	eukaryotic translation initiation factor 4 gamma 2	6			
13	*PERP*	PERP, TP53 apoptosis effector	6			
14	*ACTG1*	actin, gamma 1	5			
15	*EEF1G*	elongation factor 1-gamma	5			
16	*LGALS3*	lectin, galactoside-binding, soluble, 3	5			
17	*RPL4*	ribosomal protein L4	5			
18	*RPSA*	ribosomal protein SA	5			
19	*DCN*	decorin	5			
20	*FTH1*	ferritin, heavy polypeptide 1	5			
21	*GNB2L1*	guanine nucleotide binding protein beta2-like 1	5	X		
22	*AQP3*	aquaporin 3	5			
23	*IGHG1*	immunoglobulin heavy constant mu	5			
24	*DSP*	desmoplakin	5			
25	*TMSB4X*	thymosin, beta 4, X-linked	5			

Genes are designated by Gene Symbols, followed by descriptions and the number of clones present in the library. Genes that are known differential markers for conjunctiva, limbus, and cornea are indicated. Other genes, such as *EEF1A1* and clusterin are highly abundant in many cell types.

### Tissue markers: keratins

The most abundant tissue-specificity marker cDNA in the library is *Krt13*, ranked in abundance after eukaryotic translation elongation factor 1a1 and clusterin, which are highly abundant in many cDNA libraries. *Krt13*, represented by 13 clones, is a marker for conjunctiva [18,19] and has recently been shown to be highly expressed in limbus [20]; its partner *Krt4* (nine clones) is also in the top seven transcripts. The equivalent keratin markers for corneal epithelium are *Krt12* and *Krt3* [21]; *Krt12* is present but at lower levels (four clones), while *Krt3* does not appear in the pterygium cDNA library. This is consistent with the microarray analysis by Tong et al. [11] comparing conjunctiva and pterygium, which likewise shows that *Krt13* levels are similar in conjunctiva and pterygium and that *Krt12* is present in pterygium but not in conjunctiva.

**Figure 1:**
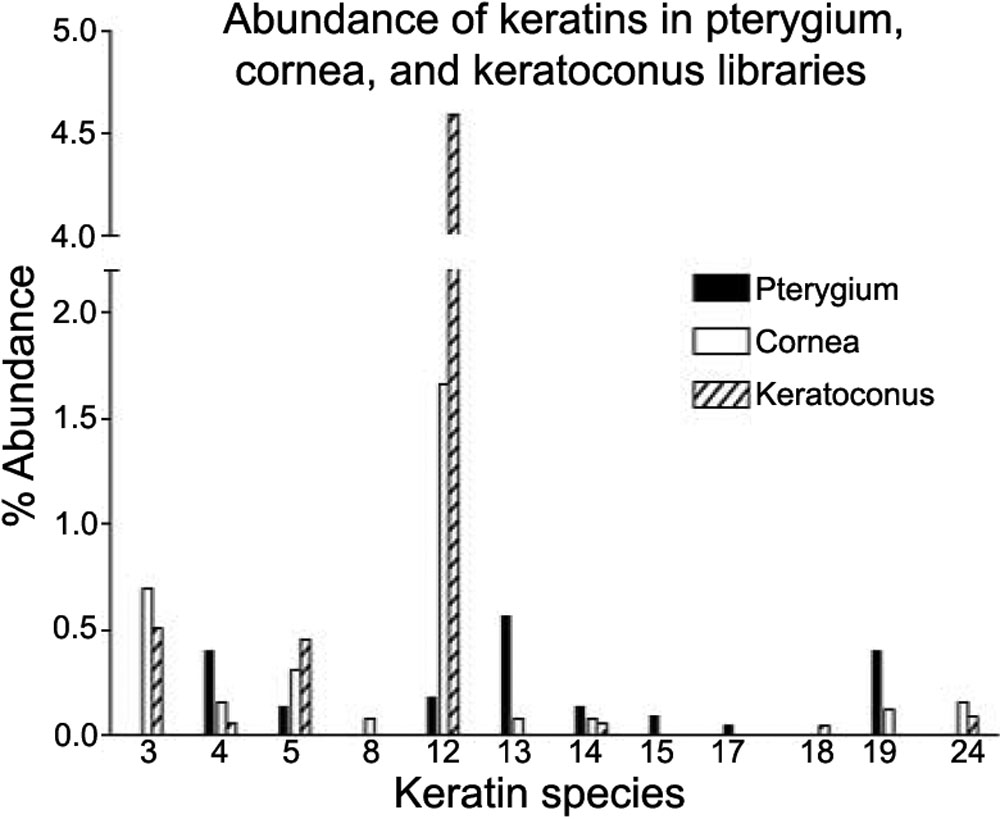
Abundance of different keratins in pterygium, cornea, and keratoconus libraries. The relative abundance of keratin transcripts in the pterygium complementary DNA library is compared to transcripts from cornea (in the unnormalized BodyMap human cornea NbLib0077) and keratoconus (in unnormalized human keratoconus cornea NbLib0073). The contents of these libraries can be viewed on the NEIBank website.

An overview of the relative abundance of all the keratin transcripts in the pterygium library in comparison with the human keratoconus (KC) cDNA library (NbLib0073) and BodyMap cornea library (NbLib0077) is shown in Figure 1. The human KC cDNA library provides a large set of cornea-derived clones that correspond well with normal cornea [22] and show the same general distribution of keratins found in microarray analysis of normal human cornea [18]. The pterygium library is represented by 2,298 clones, and there are 68,662 and 2,581 clones in the KC and BodyMap cornea libraries, respectively. Data from the only available conjunctiva library (BodyMap unnormalized human conjunctiva epithelium NbLib0108) is not included since it consists of fewer than 300 transcripts.

*Krt19* is represented by nine clones, which includes a sequence from the 3’-untranslated region. While other keratins are typically expressed as pairs (3/12; 4/13), *Krt19* does not have an obligate partner [23,24]; it is found particularly in the periderm, a transient superficial layer of developing epidermis and has been used as a marker for various tumors [25]. *Krt19* is not found among cDNAs from the KC library nor in the smaller sample of corneal epithelium cells [21], suggesting it may be a marker for pterygium. However, microarray analysis revealed significant expression of *Krt19* in conjunctiva [18]. Interestingly, *Krt19* has been proposed as a marker for limbal stem cells [26,27].

Other keratins are present in the library but not particularly abundant: the pair *Krt14* and *Krt5* both with three clones, *Krt15* with two clones, and *Krt17* with one clone. Although their representation here is small, it is interesting to note the tissue distribution of these keratins as determined by other means. Microarray analysis shows *Krt14/5* to be abundant in cornea and also, but to a lesser extent, in conjunctiva [18], while *Krt15* and *Krt17* are fivefold and 10-fold more abundant in conjunctiva, respectively. *Krt 6* does not appear in any of the human cDNA libraries, although *Krt6A* and *Krt6B* appear to be upregulated in pterygium relative to conjunctiva in microarray analysis [11]. Interestingly, there are 14 tags (1.3% of total) for *Krt6* in the NEI mouse cornea SAGE library NbLibo115 [28], suggesting that expression of *Krt6* in pterygium may occur at a low level and might be more evident with SAGE analysis.

**Figure 2:**
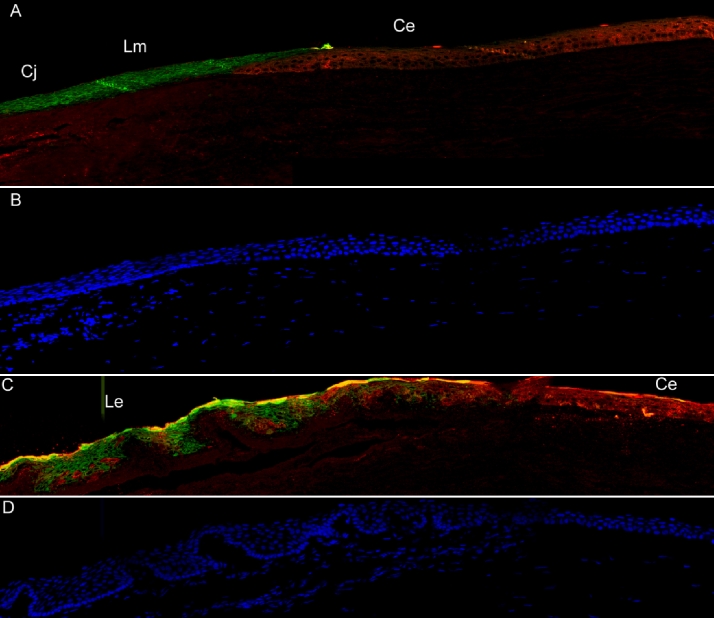
Immunofluorescence shows the localization of keratin 12 and 13 in normal ocular surface and pterygium. Krt12 is labeled in red, Krt13 in green, and nuclei are DAPI labeled in blue. **A**: Krt12 and Krt13 are segregated in corneal epithelium and conjunctival epithelium with a boundary at the limbus: Cj, conjunctiva; Lm, limbus; Ce, corneal epithelium. **B**: DAPI staining of (**A**) indicates the nuclei of conjunctiva, limbal region, and cornea. **C**: Corneal  epithelium Ce, is Krt12 positive.  The leading edge of a pterygium, Le, has mixed Krt12- and Krt13-positive cells. **D**: DAPI stain of (**C**) shows the nuclei of the leading edge region and cornea.

Immunofluorescence localization was used to examine the distribution of Krt12 and 13 in the normal ocular surface and in pterygium (Figure 2). Sections of normal human ocular surface showed a clear demarcation of these two proteins, with Krt12 staining throughout the corneal epithelium (with a fairly sharp transition at the limbus) and Krt13 staining in conjunctival and limbal epithelia (Figure 2A). A similar staining pattern for Krt13 was recently demonstrated by Ding et al. [20], who also examined the ocular surface of a normal human eye.

In the leading edge of the pterygium, there was mixed Krt12 and Krt13 staining (Figure 2C), with some cells strongly positive for one or the other marker, suggestive of a mixed population of corneal-, limbal-, and conjunctival-like cells. While Krt12 is known to be highly expressed in cornea [21], it is nevertheless relatively abundant in limbus according to microarray, quantitative Reverse Transcription PCR, and immunofluorecence studies in primates [20]. Therefore the Krt12 staining in pterygium could equally well derive from limbal cells in the leading edge. On the corneal surface, beyond the leading edge of the pterygium, Krt13 is not detectable and the normal Krt12 staining is evident. This is consistent with the EST analysis and suggests that much of the pterygium is varied in nature and that pterygium may spread across the corneal surface by migration of cells bearing conjunctival and limbal markers into the corneal epithelium.

### Other conjunctiva/cornea markers

Aquaporins also show tissue-specific distribution in the ocular surface [29], and pterygium again has markers for both conjunctiva and cornea. In pterygium, *AQP3*, predominantly a conjunctival marker, is represented by five clones. Indeed, microarray analysis does not indicate any differential expression between conjunctiva and pterygium [11]. Abundant levels of AQP3 may impact cell migration of pterygium (see below). *AQP5*, usually regarded as a corneal marker, is represented by three clones.

Uroplakin 1b has been identified as a corneal epithelium marker [21] and it is also abundant in KC [22] in cDNA library analyses. In the pterygium dataset, uroplakin 1b is represented by only one clone, while a different uroplakin-like protein (*UPLP*) gene, similar to uroplakin 3b, is abundantly represented (seven clones). Little is known about *UPLP*; the gene for human *UPLP* overlaps with a larger gene described as DNA-directed RNA polymerase II polypeptide (*POLR2J2*). *UPLP*/*POLR2J2* does not appear in cornea libraries in the NEIBank from mouse, rabbit, or human nor has it been observed in conjunctiva cDNAs [21] but may have been missed in a small sample size. However, UPLP is abundantly expressed in KC, raising the possibility that it may have a particular role in ocular surface tissues and their pathologies.

### Transcripts downregulated in pterygium relative to cornea

Several genes abundant in cornea-derived cDNA libraries are also abundant in pterygium but at lower levels than in cornea. The cornea marker *ALDH3A1* is found at a frequency of 1.4% in KC; it is the ninth most abundant clone in pterygium but it represents only 0.3% of the transcripts. Similarly, transforming growth factor β-induced, 68 kDa (*TGFBI*) accounts for 1.5% of clones in KC but only 0.13% in pterygium, an order of magnitude difference. Dysregulation of *TGFBI* expression has been implicated in numerous cancers, with effects varying according to cell type or pathological classification. For example, *TGFBI* is upregulated in renal clear cell carcinoma [30], while downregulation of *TGFBI* is causally linked to tumorigenic phenotype in both asbestos-treated [31] and radiation-exposed bronchial epithelial cells [32]. Conversely, overexpression of *TGFBI* demonstrated tumor suppressor activity in these cells [33,34]. Knockout of *TGFBI* in mice is associated with spontaneous tumor development [35]. Moreover, genetic studies have shown that missense mutations in this gene cause various forms of corneal dystrophy [36,37]. Taken together, these findings suggest that a certain minimum abundance of functional or wild-type TGFBI is necessary for maintaining normal corneal physiology. Transcripts for decorin are also among the most abundant in the pterygium cDNA library, and it, too, is reduced in relative abundance compared with KC; it is at 1.5% in KC but only 0.2% in pterygium. Reduced decorin expression is similarly associated with tumorigenic phenotypes [38], while increased levels are suppressive [39-41]. As is the case with *TGFBI*, mutations in the decorin gene are responsible for corneal dystrophy [42]. In cornea, decorin has been shown to interact with TGFBI in complexes that in turn bind collagen type VI [43], suggesting these proteins, both of which are expressed at lower levels in pterygium, work together in maintaining normal cornea.

Decreased expression of decorin is paralleled by decreased expression of several closely related proteins. Decorin is a member of the small leucine-rich proteoglycan (SLRP) family of proteins. Other members of this family, keratocan, lumican, and mimecan (also called osteoglycin) are also highly expressed in cornea and are similarly thought to contribute to corneal transparency [44-47]. In an unnormalized rabbit cornea library (NbLib0086), lumican, keratocan, and osteoglycin account for 1.3, 1.3, and 1.4% of transcripts, respectively. In contrast, there are no transcripts for any of these SLRPs in the pterygium library. It is interesting that the SLRP family genes, so important to corneal transparency, are collectively downregulated in pterygium.

### Other abundant transcripts

**Figure 3:**
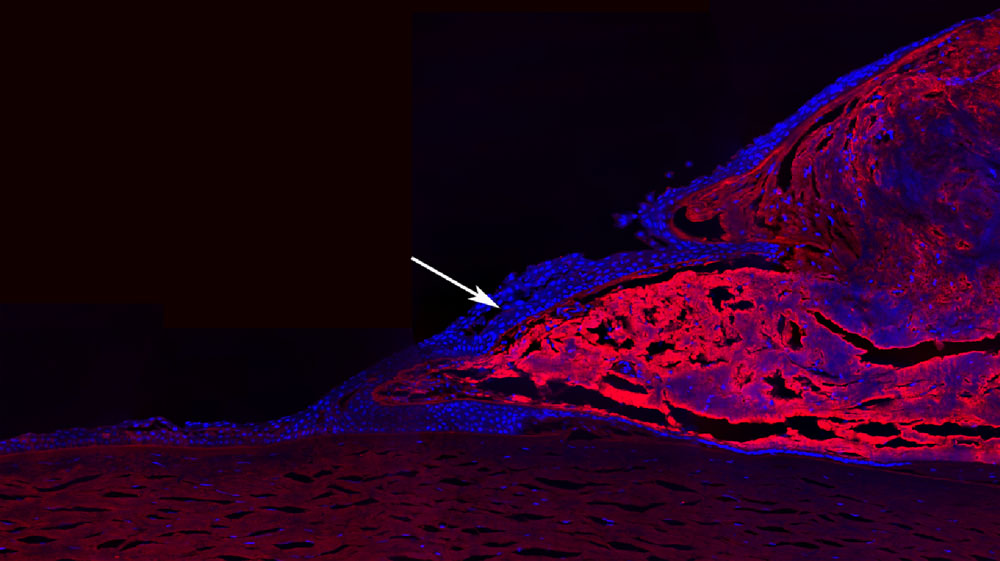
The abundance of clusterin in pterygium is illustrated by immunofluorecence. Labeling for clusterin is in red, nuclei are blue (DAPI). The arrow indicates cells enveloping the body of the pterygium that are clusterin negative.

One of the most abundantly expressed genes in pterygium (14 clones) is that encoding clusterin, which is highly expressed in cornea and other ocular tissues [21,22]. As implied by the existence of numerous other names (apolipoprotein J, testosterone-repressed prostate message 2, sulfated glycoprotein 2, complement-associated protein SP-40), clusterin is a multifunctional protein whose role in pterygium is open to speculation. Immunofluorescence labeling of clusterin in pterygium showed an interesting pattern (Figure 3). There was intense staining for clusterin throughout the fibrovascular body of the pterygium, but expression was much lower (or absent) in the outer layer of cells, particularly at the leading edge. This suggests that the pterygium body is enveloped by cells with a different gene expression profile from the internal mass of cells.

Transcripts from the immunoglobulin κM light-chain locus (13 clones) on chromosome 2p11.2 are abundant in the pterygium dataset. There are also multiple clones for immunoglobulin μ constant regions. This suggests the presence of high levels of immunoglobulin M (IgM) in pterygium samples. κM chains in particular have been associated with autoimmune reaction [48], while high overall levels of IgM may reflect local reaction to a pathogen in one or more of the pterygium donors. Interestingly, IgM transcripts are also abundant in human lacrimal gland [49], suggesting that populations of IgM-expressing cells may be commonly present at the ocular surface.

Although EMT has been implicated in the etiology of pterygium [1,12], genes specifically annotated with EMT associations in gene ontology [50] are not notably abundant in the pterygium cDNA library. Those that are so annotated are *S100A4* (two clones), bone morphogenetic protein 7 (one clone), heterogeneous nuclear ribonucleoprotein A/B (one clone), and alanine-glyoxylate aminotransferase 2-like 2 (one clone). One gene abundantly expressed in pterygium, *LGALS3*/galectin-3 (five clones), has been associated with human fibrotic liver disease [51] and thus might play a similar fibrosis-related role in pterygium. However *LGALS3*/galactin-3 is widely expressed and is found in ocular tissues as different as normal lens and lacrimal gland. Although MMPs have been shown to be upregulated in pterygium tissue [10], they are not abundant enough to be represented in the library.

### Migration markers

**Figure 4:**
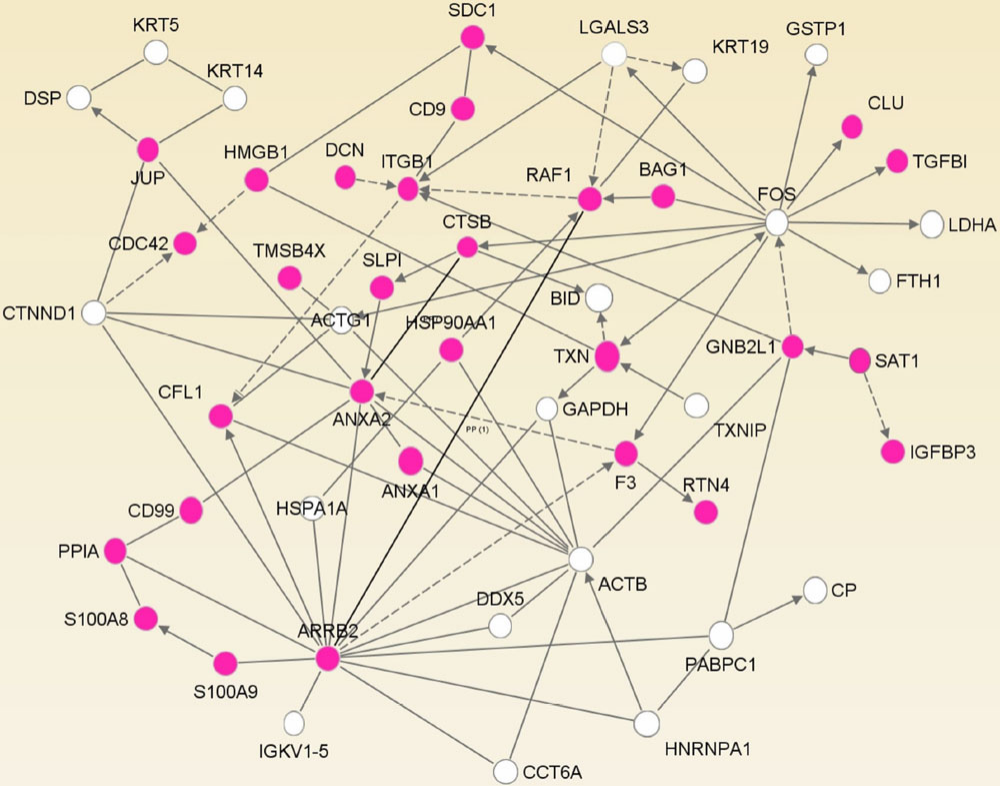
Network of abundant genes in the pterygium cDNA library is dominated by functional associations with cell migration processes. Genes represented by three or more transcripts in the pterygium cDNA library were analyzed by Ingenuity Pathway Analysis. Each gene (node) is represented by a circle and labeled with its official gene symbol. Direct and indirect relationships are indicated by solid and dotted lines, respectively. Arrows indicate the direction of intermolecular action. Genes associated with cell migration are shown in color.

In addition to the genes that are markers for ocular surface cell types, pterygium cDNA analysis reveals several abundantly expressed genes that have roles in cell migration. This is illustrated by the network shown in Figure 4. The network was generated using IPA to draw connections between genes in the pterygium dataset represented by three or more clones, based upon biological relationships. Some major hubs are evident, most notably β2-arrestin (*ARRB2*), c-Fos (*FOS*), β-actin (*ACTB*), and annexin A2 (*ANX2*). Unconnected genes were removed from further consideration. Functional analysis of the network showed that 29 of the 51 genes are associated with cell migration (p value of 8.10E-20).

Notable among this class of migration-related transcrits is spermidine/spermine N1-acetyltransferase 1 (*SAT1*), represented by nine clones. SAT1 is a highly regulated rate-limiting enzyme in polyamine metabolism [52] and as such has roles in many cellular responses. Among these, and of interest in pterygium, is integrin α9-dependent cell migration [53]. Overexpression of *SAT1* due to gene duplication has in addition been implicated in keratosis follicularis spinulosa decalvans [54], a disease that includes corneal degeneration. Among many other functions, SAT1 has been shown to be involved in the oxygen-independent degradation of hypoxia-inducible factor 1α (HIF-1α) through interaction with both HIF-1α and RACK1/guanine nucleotide-binding protein β2-like 1 (GNB2L1) [55]. Interestingly, RACK1/GNB2L1 also appears in the list of most abundant pterygium cDNAs (Table 1), so the potential for SAT1/GNB2L1 complex formation exists.

**Figure 5:**
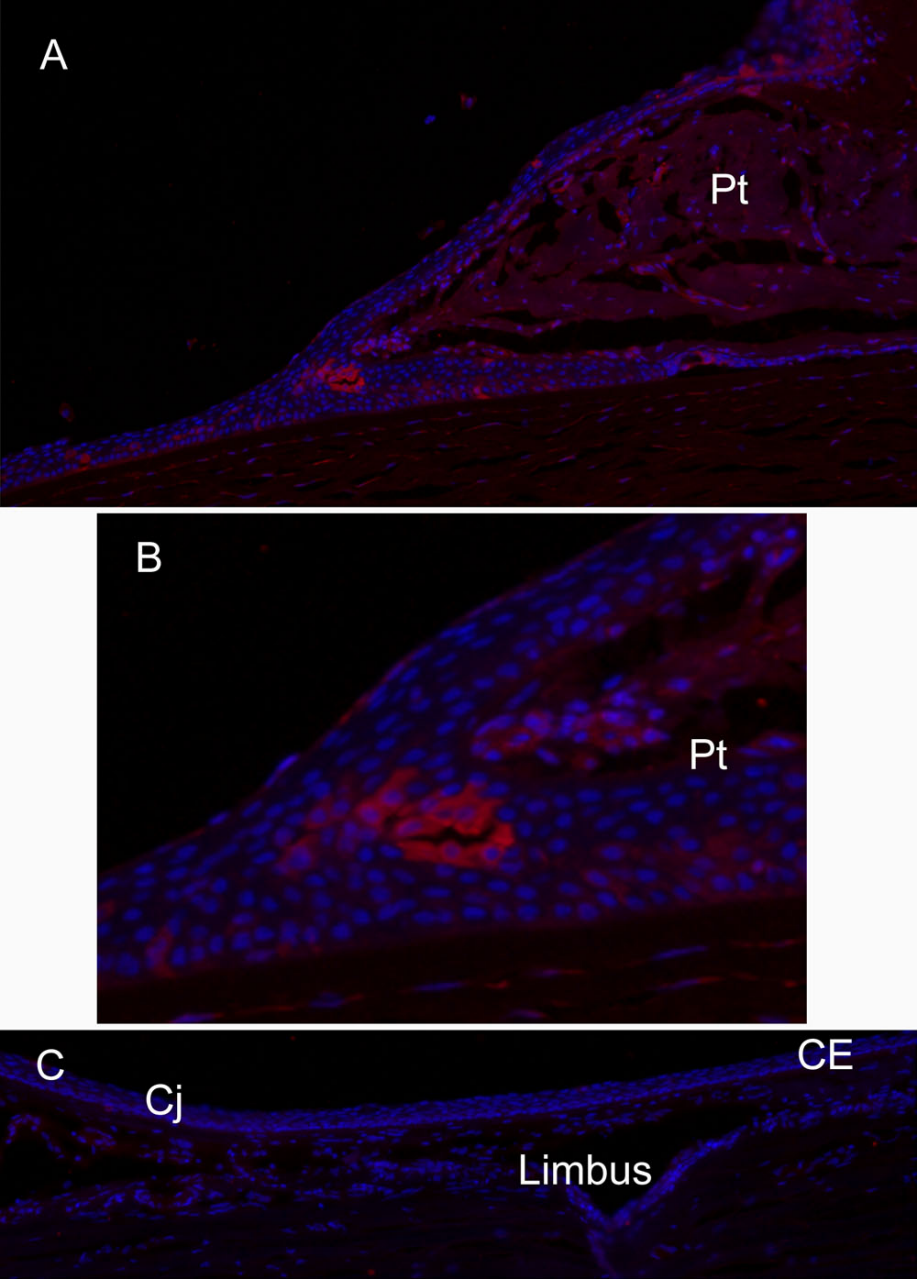
Immunofluorescence indicates that SAT1 expression is associated with the leading edge and body of pterygium. SAT1 immunofluorescence (IF) is shown in red and nuclei are DAPI labeled in blue. CE, corneal epithelium; Pt, pterygium; Cj, conjunctiva. **A**:  SAT1 is detectable throughout the body of the pterygium with a strongly labeled clump of cells at the leading edge. **B**: Magnified view is presented of the clump of cells strongly positive for SAT1 at the apex of the pterygium body. **C**: No SAT1 IF is apparent in limbus and normal cornea from the other side of the same eye (section torn in corneal stroma).

The distribution of SAT1 in normal cornea and pterygium was examined by immunofluorescence (Figure 5). Staining for SAT1 was weak or absent in normal corneal epithelium and limbus but, consistent with its cDNA abundance, was detectable throughout the body of the pterygium. Some intensely staining groups of cells were observed, particularly at the leading edge of the pterygium body (Figure 5B).

**Figure 6:**
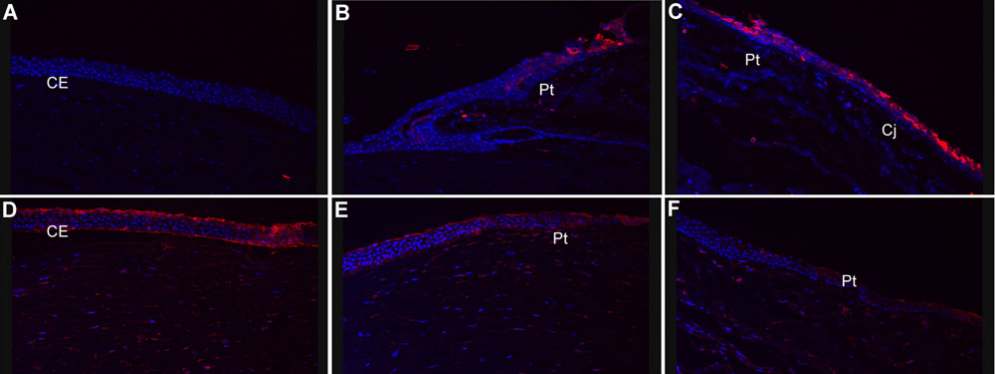
Immunfluorescence of SA100A9 and A6 shows differential expression in pterygium and normal cornea. S100A9 and S100A6 immunfluorescence are shown in red, and nuclei are DAPI labeled in blue. CE, corneal epithelium; Pt, pterygium; Cj, conjunctiva. Panels **A**–**C** show results for S100A9, and panels **D**–**F** show results for S100A6. **A**: S100A9 in normal corneal epithelium. **B**: S100A9 in pterygium body. **C**: S100A9 in pterygium/conjunctiva boundary. **D**: S100A6 in normal corneal epithelium. **E**: S100A6 in pterygium leading edge. **F**: S100A6 in pterygium body. SA100A9 is clearly detectable in the conjunctiva and in the pterygium but is absent from normal corneal epithelium while SA100A6 is most abundant in corneal epithelium and is at much lower levels in pterygium and conjunctiva.

Annexin A2, a network hub in Figure 4, is represented by seven clones in the pterygium collection. It is a calcium-dependent membrane-binding protein that is implicated in endocytosis [56] and also in epithelial cell migration [57]. Interestingly, annexin A2 has been shown to play a role in the induction of MMP-9 activity [58], a hallmark of pterygial cell migration [9]. Annexin A2 can form heterotetramers with the small calcium-binding protein p11/S100A10 [56], but no clones for *S100A10* are present among those sequenced from the pterygium library. However, a different member of the S100 family, *S100A9* (also known as calgranulin B or *MRP14*) is abundant in pterygium and is represented by10 clones. S100A9 is not known to be abundant in cornea and is absent from the KC cDNA collection. SA100A9 is implicated in inflammation and is upregulated in psoriasis [59]. It may also be involved in control of cell migration since antibodies to S100A9 block migration of tumor cells [60]. Other members of the S100 family, A4, A6, A8, A11, and A14, are also represented in the pterygium cDNA library. Immunofluorescence using antibodies for A9 (Figure 6A–C) and A6 (Figure 6D–F) revealed an interesting distinction between pterygium and normal cornea. S100A9 was detectable in regions of the pterygium body and in the pterygium/conjunctiva region, with no detectable labeling in normal corneal epithelium (Figure 6A), consistent with EST data. In contrast, S100A6 was detectable in normal corneal epithelium but not in pterygium. This also corresponds to the EST data; levels of abundance for S100A6 in the pterygium and BodyMap cornea libraries were 0.04% and 0.7%, respectively. Although a recent study of S100 proteins in pterygium and conjunctiva shows significant expression of A6 and A9 in both tissues, the degree of difference between pterygium and cornea is not addressed [61]. The data presented here suggest that S100A9 may be a pterygium and/or conjunctiva marker.

Several other genes associated with cell migration or neoplastic phenotypes are also represented by multiple clones in the pterygium dataset. There are three clones for Jagged 1, which is mutated in Alagille syndrome [62] and is a major ligand for Notch proteins. Notch/Jagged interaction is associated with tumorigenesis and proliferation and also with epithelial-to-mesenchymal transition [63]. Tumor protein 63 (*Tp63*), a transcriptional regulator related to p53, is commonly overexpressed in squamous carcinomas of epithelium [64] and is represented by two clones. Tp63 has been shown to regulate expression of several classes of genes involved in cell migration, among them AQP3 [55], which has been shown to be required for cell migration and proliferation during corneal re-epithelialization [65]. As noted above, AQP3 itself is represented by five clones in the pterygium dataset.

### Effect of modulation of SAT1 upon migration of pterygium primary cultures

**Figure 7:**
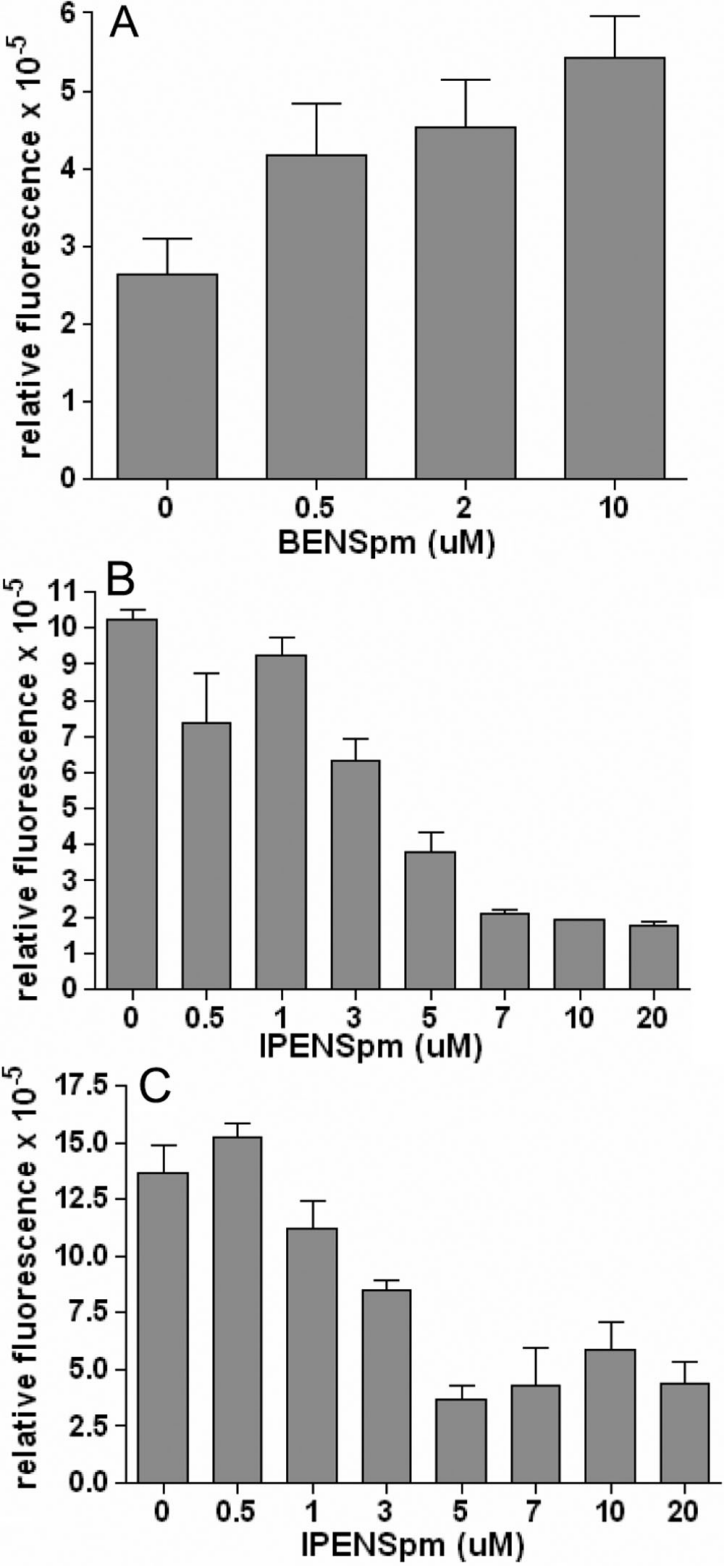
Effect of polyamine analogues on migration of primary cultures of pterygium cells. Plots show the relative migration of cells treated with either **A**: BENSpm (no serum), **B**: IPENSpm (no serum), or **C**: IPENSpm (5% serum). Migration was assayed in terms of the fluorescent signal at 480/520 nm. Error bars indicate the standard error of the mean. Each set of conditions was tested a minimum of four times.

The relative abundance in pterygium of transcripts for certain proteins associated with cell migration suggests pathways that may be important in the spread of pterygium. Targeted inhibition of some of these proteins could therefore be useful in reducing the progression of the growth. SAT1, which is among the most abundant transcripts in pterygium and which, by immunofluorescence, appears to be at higher levels in pterygium than in normal cornea, is a potential target for such inhibition. In addition to its central role in many cellular processes through regulation of polyamine levels and its complex patterns of upregulation and downregulation in various disease states, SAT1 has also been shown to enhance cell migration through specific binding to integrin α9 [20]. This effect requires that the catalytic machinery of SAT1 be functional, suggesting local effects on polyamines may be important modifiers of SAT1–integrin-dependent cell migration. Numerous polyamine analogues have been developed, particularly for use as antitumor agents. Several of these act as inhibitors of SAT1 activity [66-68] but depending on the particular cell type involved may produce different results [69]. The analogue BENSpm superinduces SAT1 [70,71], while other analogues, such as IPENSpm, have only minor effects on SAT1 [72]. We tested the effects of these two compounds on the migration of cultured pterygium cells (Figure 7). In tests under serum-free conditions with concentrations up to 10 μM, BENSpm produced an increase in migration (Figure 7A). In contrast, over a similar concentration range, IPENSpm caused a significant inhibition of cell migration (Figure 7B). The inhibitory effect of IPENSpm was also investigated in the presence of 5% serum (Figure 7C). A significant reduction in cell migration was again observed, with a maximum effect at a concentration of 5μM. Although several polyamine analogues (notably BENSpm) have been shown to have cytotoxic effects on various tumor cell lines, neither drug had any effect upon cultured pterygium cell viability when tested at 10 µM concentrations (data not shown). Although a higher concentration (20 μM) of IPENSpm was also tested in migration assays, the results were not substantially different from those obtained at 10 μM concentrations; thus it is unlikely that toxicity played a role in migration results.

### Conclusions

EST analysis of excised human pterygia shows that differentiation markers for conjunctiva, limbus, and cornea are present, with conjunctival and limbal markers more abundantly expressed. This is illustrated by the higher abundance of clones for *Krt13/4* in pterygium compared with the smaller number of clones for the corneal marker *Krt12* and the absence of its partner *Krt3*. Immunofluorescence localization shows a mixed population of *Krt13-* and *Krt12*-positive cells in the leading edge, supporting the paradigm of pterygium as an invasion of the cornea by altered limbal cells, followed by conjunctival cells [9]. At the same time that conjunctival and limbal markers predominate, expression of some genes characteristic of cornea is diminished in the pterygium cDNA library. These include the cornea marker *ALDH3A1* and the SLRPs, a family of proteins highly expressed and functionally important in the cornea.

Pathway analysis calls attention to connections among genes that are abundant in the pterygium cDNA dataset and that share an association with cell migration. Indeed, immunofluorescence data are consistent with migration of altered cells into the corneal epithelium. One of these genes, *SAT1*, is a key regulator of polyamines, and one polyamine analogue, IPENSpm, a potential inhibitor of SAT1, significantly reduces migration in primary cultures of pterygium. Inhibitors of cell migration, such as IPENSpm, may provide new opportunities for therapeutic approaches to block or reduce the spread of pterygium.
